# Positive and Negative Selection in Transgenic Mice Expressing a T-Cell Receptor Specific for Influenza Nucleoprotein and Endogenous Superantigen

**DOI:** 10.1155/1993/98015

**Published:** 1993

**Authors:** Clio Mamalaki, James Elliott, Trisha Norton, Nicholas Yannoutsos, Alain R. Townsend, Phillip Chandler, Elizabeth Simpson, Dimitris Kioussis

**Affiliations:** 1 Laboratory of Molecular Immunology National Institute for Medical Research The Ridgeway Mill Hill London NW7 1AA UK; 2 Institute of Molecular Medicine John Radcliffe Hospital Headington Oxford OX3 9DU UK; 3 Clinical Research Centre Watford Road Harrow Middlesex HA1 3UJ UK

**Keywords:** F5 TcR transgenic mice, Vα4, Vβ11, MHC restriction, Mls

## Abstract

A transgenic mouse was generated expressing on most (>80%) of thymocytes and peripheral T cells a T-cell receptor isolated from a cytotoxic T-cell clone (F5). This clone is CD8^+^ and recognizes αα366-374 of the nucleoprotein (NP 366-374) of influenza virus (A/NT/60/68), in the context of Class ,MHC D^b^ (Townsend et al., 1986). The receptor utilizes the Vβ11 and Vα4 gene segments for the β chain and α chain, respectively (Palmer et al., 1989). The usage of Vβ11 makes this TcR reactive to Class II IE molecules and an endogenous ligand recently identified as a product of the endogenous mammary tumour viruses (Mtv) 8, 9, and 11 (Dyson et al., 1991). Here we report the development of F5 transgenic T cells and their function in mice of the appropriate MHC (C57BL/10 H-2^b^, IE^-^) or in mice expressing Class II MHC IE (e.g., CBA/Ca H-2^k^ and BALB/c H-2^d^) and the endogenous Mtv ligands. Positive selection of CD8^+^ T cells expressing the Vβ11 is seen in C57BL/10 transgenic mice (H-2^b^). Peripheral T cells from these mice are capable of killing target cells in an antigen-dependent manner after a period of ^in vitro^ culture with IL-2. In the presence of Class II MHC IE molecules and the endogenous Mtv ligand, most of the single-positive cells carrying the transgenic T-cell receptor are absent in the thymus. Unexpectedly, CD8^+^ peripheral T-cells in these (H-2^k^ or H-2^d^) F5 mice are predominantly Vβ11 positive and also have the capacity to kill targets in an antigen-dependent manner. This is true even following backcrossing of the F5 TcR transgene to H-2^d^ scid/scid mice, in which functional rearrangement of endogenous TcR alpha- and beta-chain genes is impaired.


*Developmental Immunology*, 1993, Vol. 3, pp. 159–174
Reprints available directly from the publisher
Photocopying permitted by license only

© 1993 Harwood Academic Publishers GmbH
Printed in the United States of America

### Positive and Negative Selection in Transgenic Mice Expressing a T-Cell Receptor Specific for Influenza Nucleoprotein and Endogenous Superantigen

CLIO MAMALAKI,† JAMES ELLIOTT,† TRISHA NORTON,† NICHOLAS YANNOUTSOS,†
ALAIN R. TOWNSEND,‡ PHILLIP CHANDLER,§ ELIZABETH SIMPSON,§ and DIMITRIS KIOUSSIS*†

†*Laboratory of Molecular Immunology, National Institute for Medical Research, The Ridgeway, Mill Hill, London NW7 1AA, U.K.*
‡*Institute of Molecular Medicine, John Radcliffe Hospital, Headington, Oxford, OX3 9DU, U.K.*
§*Clinical Research Centre, Watford Road, Harrow, Middlesex, HA1 3UJ, U.K.*

A transgenic mouse was generated expressing on most (>80%) of thymocytes and peripheral T cells a T-cell receptor isolated from a cytotoxic T-cell clone (F5). This clone is CD8$^+$ and recognizes $\alpha\alpha$366-374 of the nucleoprotein (NP 366-374) of influenza virus (A/NT/60/68), in the context of Class I MHC D$^b$ (Townsend et al., 1986). The receptor utilizes the V$\beta$11 and V$\alpha$4 gene segments for the $\beta$ chain and $\alpha$ chain, respectively (Palmer et al., 1989). The usage of V$\beta$11 makes this TcR reactive to Class II IE molecules and an endogenous ligand recently identified as a product of the endogenous mammary tumour viruses (Mtv) 8, 9, and 11 (Dyson et al., 1991). Here we report the development of F5 transgenic T cells and their function in mice of the appropriate MHC (C57BL/10 H-2$^b$, IE$^-$) or in mice expressing Class II MHC IE (e.g., CBA/Ca H-2$^k$ and BALB/c H-2$^d$) and the endogenous Mtv ligands. Positive selection of CD8$^+$ T cells expressing the V$\beta$11 is seen in C57BL/10 transgenic mice (H-2$^b$). Peripheral T cells from these mice are capable of killing target cells in an antigen-dependent manner after a period of *in vitro* culture with IL-2. In the presence of Class II MHC IE molecules and the endogenous Mtv ligand, most of the single-positive cells carrying the transgenic T-cell receptor are absent in the thymus. Unexpectedly, CD8$^+$ peripheral T-cells in these (H-2$^k$ or H-2$^d$) F5 mice are predominantly V$\beta$11 positive and also have the capacity to kill targets in an antigen-dependent manner. This is true even following backcrossing of the F5 TcR transgene to H-2$^d$ scid/scid mice, in which functional rearrangement of endogenous TcR alpha- and beta-chain genes is impaired.

KEYWORDS: F5 TcR transgenic mice, V$\alpha$4, V$\beta$11, MHC restriction, Mls.

### INTRODUCTION

Development and differentiation of T cells are complex processes taking place mainly in the thymus. Stem cells originating in the fetal liver or the adult bone marrow migrate into the thymus, where they mature, become functionally competent and are finally exported into the peripheral lymphoid compartment (Adkins et al., 1987). The majority (>95%) of thymocytes and circulating T cells carry the $\alpha/\beta$ TcR heterodimer derived from the somatic rearrangement of the TcR alpha and beta loci genes, whereas a small proportion (2–5%) carries the $\gamma/\delta$ TcR heterodimer derived from similar rearrangement of the TcR $\gamma$ and $\delta$ loci (Davis and Bjorkman, 1988). The T-cell receptor (TcR) is the structure that determines the antigenic specificity of the T cell and the repertoire is selected for the ability of the T-cell receptor to recognize self-MHC molecules complexed or not with peptide. The development of self-MHC restriction and self-tolerance is the result of two major mechanisms: positive selection, during which potentially functional cells able to recognize peptides presented by self-MHC molecules are allowed to mature, and negative selection, during which potentially harmful or self-reactive cells are eliminated (Blackman et al., 1990; von Boehmer, 1990; Loh,

*Corresponding author.

1991). The difference between these two similar but contradictory processes may be based on requirements for higher-affinity interaction between components of the TcR/MHC/peptide complex for negative selection than for positive selection (Sprent et al., 1988; Schwartz, 1989). It is generally thought that thymic epithelial cells positively select those thymocytes that have a TcR with affinity for self-MHC, whereas negative selection is carried out following presentation of self-antigens by bone marrow-derived thymic stroma cells (Sprent et al., 1975; Bevan and Fink, 1978; Matzinger and Guerder, 1989; Speiser et al., 1989; Mazda et al., 1991).

In mice, monoclonal antibodies specific for different TcR V$\beta$ chains allow detection of percentages of different V$\beta$ positive thymocytes in the $CD4^{+}8^{+}$ compartment in comparison with their $CD4^{+}$ and $CD8^{+}$ single positive progeny. Thus, mice showing deletion of peripheral T cells with a particular V$\beta$ chain have V$\beta$-positive cells within the $CD4^{+}8^{+}$ double-positive but not in the $CD4^{+}$ or $CD8^{+}$ single-positive thymocyte compartments. This approach, using V$\beta$-specific antibodies, provided the first evidence for discrete but measurable negative selection events (Kappler et al., 1987, 1988; MacDonald et al., 1988), and since then has provided evidence for V$\beta$-specific positive selection in certain circumstances (Blackman et al., 1989, 1990; Tomonari, 1992).

The use of T-cell receptor transgenic mice has provided another extremely powerful way of examining both positive and negative selection because these mice carry a specific T-cell receptor $\alpha/\beta$ heterodimer on most of their thymocytes and mature T cells, thus allowing easy detection of their fate (Kisielow et al., 1988a,b; Sha et al., 1988a; Berg et al., 1989; Kaye et al., 1989; Pircher et al., 1989).

These studies have demonstrated similarities between the different systems, such as the positive selection in the presence of the appropriate MHC haplotype (Teh et al., 1989; Ohashi et al., 1990) and the deletion of developing thymocytes in the presence of the cognate antigen (Berg et al., 1989; Scott et al., 1989). They also revealed some disparities. Thus, male mice carrying a TcR recognizing the H-Y antigen delete thymocytes at the immature double-positive stage (Kisielow et al., 1988a) whereas mice transgenic for TcRs reacting to $Mls^{a}$ seem to delete single-positive self-reacting cells (Pircher et al., 1989). It is not clear at the moment to what these differences are due. Analysis of more transgenic mice with different TcRs specific for a variety of antigens should illuminate these disparities and help to elucidate basic mechanisms.

In order to study the phenomena governing selection mechanisms, we generated a C57BL/10 ($H\text{-}2^{b}$) transgenic mouse carrying a specific T-cell receptor $\alpha$ and $\beta$ cDNAs under the control of CD2 promoter and Locus Control Region (LCR). The majority of thymocytes and T cells in these mice express the T-cell receptor from a cytotoxic T-cell clone that recognizes a peptide from influenza nucleoprotein in the context of H-2 $D^{b}$. Here we describe positive and negative selection occurring in transgenic mice with appropriate MHC haplotype or in mice expressing Mtv products that are recognized by the transgenic receptor.

### RESULTS

### Generation of Transgenic Mice Carrying the F5 T-Cell Receptor

In order to generate the constructs for microinjection, we inserted the cDNAs for the $\alpha$ and $\beta$ chains of the F5 cytotoxic clone is an artificial Eco RI site at the 5' untranslated region of a human CD2 minigene (Robey et al., 1992) that has a frameshift mutation in the CD2 coding region (Fig. 1A). This CD2 minigene cassette provides an intron, a polyadenylation signal, and the human CD2 3' regulatory (LCR) sequences, which are sufficient to direct expression of transgenes in thymocytes and T cells of transgenic mice in a tissue-specific, gene-copy-related and position-independent manner (Greaves et al., 1989).

The chimeric $\alpha$ and $\beta$ genes were cointroduced by egg microinjection in the genome of C57BL/10 mice, as described in Lang et al. (1988). Several lines were generated that carried and expressed the transgenes. We analyzed in detail a line that contained a high number of copies of both the $\alpha$- and $\beta$-chain genes, as shown by Southern blot analysis of tail DNA (Fig. 1B). Thus, on a Southern blot of SacI digested genomic DNA, hybridized with a human CD2 cDNA probe, we observed three bands of 2.3, 2.5, and 1.8 kb corresponding to the F5 $\alpha$ TcR-CD2, F5$\beta$TcR-CD2, and a common 3' CD2 fragment, respectively (Fig. 1B).

In order to determine whether the integrated hybrid transgenes could be transcribed into mRNA containing F5 TcR $\alpha$- and $\beta$-specific sequences, Northern blot analysis was performed on total RNA isolated from thymus of F5 TcR transgenic mice (Fig. 1B). Hybridization of the blots to V$\alpha$4- and V$\beta$11-specific DNA probes showed that both transgenes were transcribed in the thymus of the transgenic mice giving rise to mainly one band of approximately 3 kb representing a hybrid TcR-CD2 transcript that is larger than the normal mouse TcR $\alpha$ and $\beta$ mRNAs due to the hybrid nature of the transcriptional unit (Fig. 1A).

### Surface Expression of the Transgenic T-Cell Receptor in Thymus and Lymph Node of Transgenic Mice

In order to follow the surface expression of the F5 T-cell receptor, fluorometric (FACS) analysis of thymus and lymph node cells of transgenic mice was performed. Thymocytes and lymph node cells were stained with KT11, a monoclonal antibody that is specific for T-cell receptors using the V$\beta$11 gene segment (Tomonari and Lovering, 1988). Over 80% of thymocytes of transgenic mice were found to express the V$\beta$11 chain, compared with 1–4% of thymocytes from nontransgenic littermates (Fig. 2A). Two distinct populations of thymocytes with different densities of V$\beta$11 TcR chain could be distinguished in the thymus of

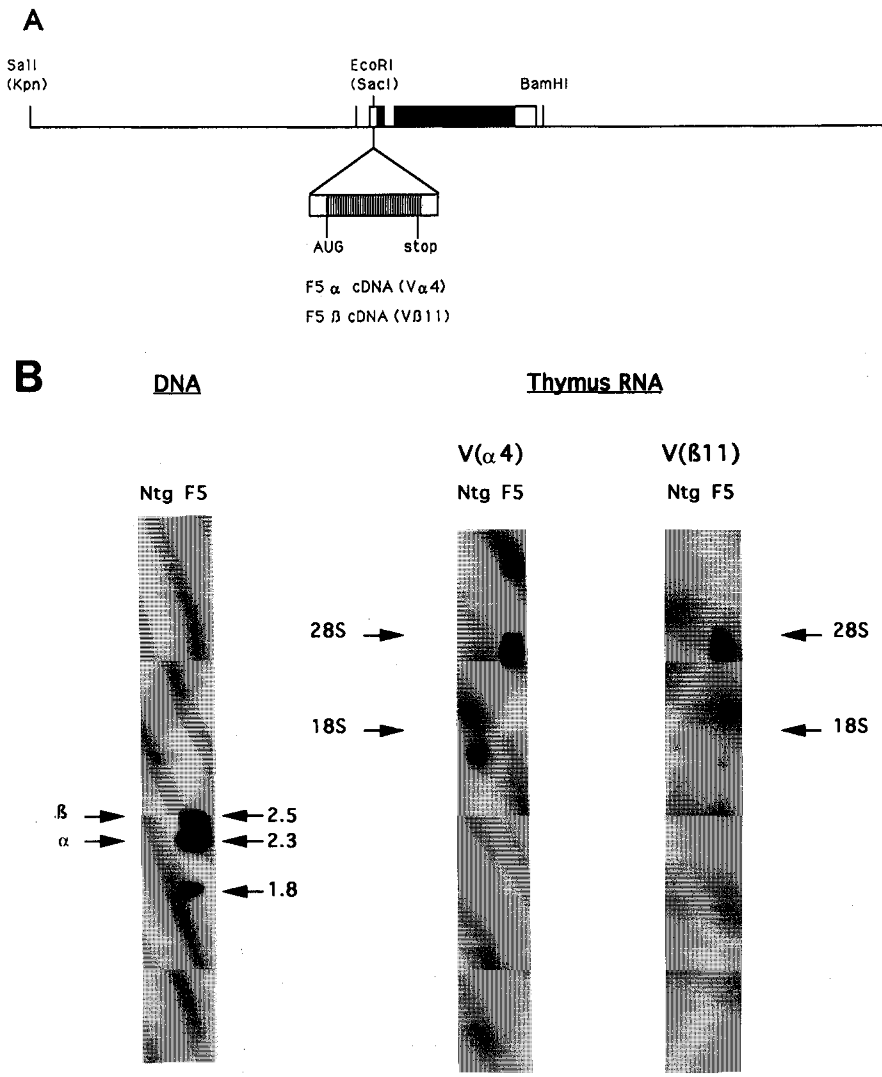

FIGURE 1. Southern and Northern blot analysis of F5 transgenic mice. (A) Schematic representation of the fragments used for microinjection. The solid box indicates the hCD2 cDNA and the striped box the cDNAs for F5$\alpha$ or F5$\beta$ chains and the lines indicate hCD2 5' and 3' flanking genomic sequences. (B) DNA from tail biopsy (5 mg) from F5 transgenic and nontransgenic control mice was hybridized to a hCD2 cDNA probe. Total RNA (10 mg) from thymuses of F5 transgenic and nontransgenic control mice were hybridized to probes specific for the V$\alpha$4 and V$\beta$11 gene segments.

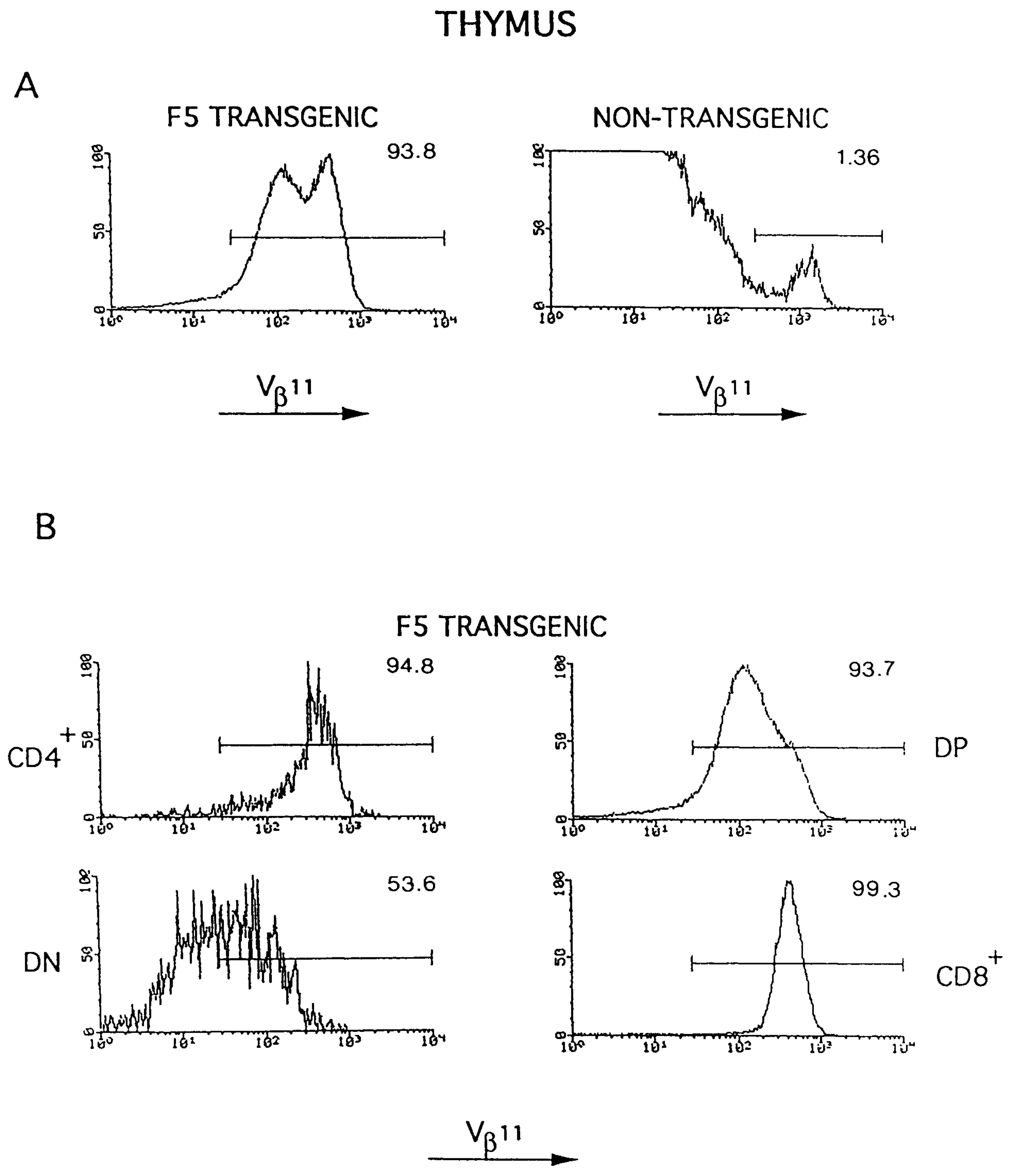

FIGURE 2. Expression of $V\beta 11$ on thymocytes and lymph node cells from F5 TcR transgenic and control nontransgenic mice. Thymocytes from F5 transgenic or control nontransgenic mice were stained with FITC-conjugated anti-CD8, anti-CD4-RED613$^R$, and KT11 anti-$V\beta 11$ antibody, followed by phycoerythrin-conjugated streptavidin. $V\beta 11$ expression (A) on total thymocytes from F5 TcR transgenic and nontransgenic mice; (B) on gated $CD4^-CD8^-$(DN), $CD4^+8^+$(DP), $CD4^+8^-$ ($CD4^+$), $CD4^-CD8^+$ ($CD8^+$) thymic subpopulations.

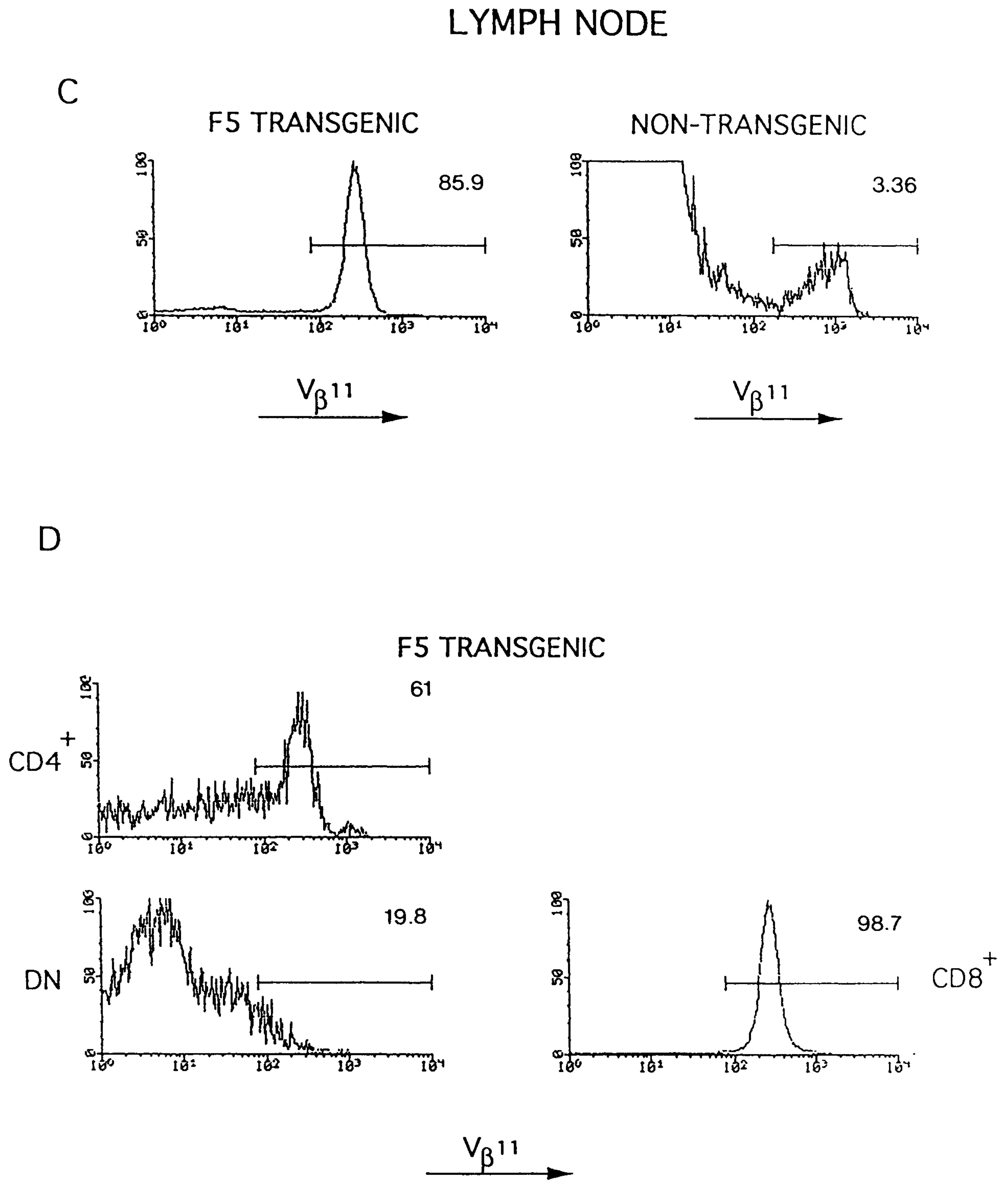

FIGURE 2. Expression of V$\beta$11 on thymocytes and lymph node cells from F5 TcR transgenic and control nontransgenic mice. Thymocytes from F5 transgenic or control nontransgenic mice were stained with FITC-conjugated anti-CD8, anti-CD4-RED613$^R$, and KT11 anti-V$\beta$11 antibody, followed by phycoerythrin-conjugated streptavidin. V$\beta$11 expression; (C) on total lymph node cells from F5 TcR transgenic and nontransgenic control mice stained as before; and (D) on different subpopulations of lymph node cells.

transgenic mice. One population with low levels of TcRαβ expression ($TcR^{lo}$) and a population expressing the Vβ11 TcR chain at levels that were intermediate ($TcR^{int}$) between this low ($TcR^{lo}$) level of expression and the high ($TcR^{hi}$) levels found on mature single-positive thymocytes of nontransgenic mice (Fig. 2A).

To follow the expression of the transgenic T-cell receptor in different subpopulations of thymocytes, we performed three-color fluorometric analysis using antibodies recognizing CD4, CD8, and Vβ11 TcR molecules (Fig. 2B). The majority of $CD4^+8^+$ and 20–50% of $CD4^-8^-$ thymocytes express low levels of the Vβ11 TcR chain. The majority (>90%) of $CD8^+4^-$ thymocytes express the intermediate levels of Vβ11 TcR chain, whereas a smaller proportion (70–90%) of the $CD8^-4^+$ single-positive cells were Vβ11-positive.

In Figs. 2C and 2D, similar analysis of lymph node cells from F5 transgenic mice showed that over 95% of $CD8^+CD4^-$ cells were expressing Vβ 11, whereas a significant proportion of $CD4^+CD8^-$ cells (40–60%) were Vβ11 negative. It is not clear which Vβ gene segment these $CD4^+V\beta11^-$ cells are expressing. There was a small population of $CD4^+8^-$ lymph node T cells expressing a high level of Vβ11. No significant expression of Vβ11 was found in the $CD4^-8^-$ cell population in the lymph nodes (Fig. 2D).

### Positive Selection in F5 Transgenic Mice

In order to study the effect of the F5 transgenic receptor expression on the $CD4^+/CD8^+$ ratio, thymocytes and lymph node cells from the F5 mice were stained with anti-CD4 and anti-CD8 antibodies. In F5 transgenic mice, the proportion of $CD8^+4^-$ thymocytes was significantly increased when compared to nontransgenic littermates from about 2% in nontransgenic mice to about 8–30% in F5 transgenic mice (Figs. 3 and 4C). A concomitant decrease in the proportion of $CD4^+8^-$ mature thymocytes was observed from about 9% in nontransgenic to about 2% in F5 transgenic mice (Fig. 3).

Similarly, a pronounced increase in the proportion of $CD8^+4^-$ cells was observed in the lymph nodes of transgenic mice (Fig. 3). Thus, approximately 70% of the transgenic lymph node cells were of $CD8^+4^-$ phenotype, whereas in nontransgenic lymph nodes, the proportion of $CD8^+4^-$ cells was 25%. This represents a change in the ratio of $CD8^+/CD4^+$ cells from 0.5 in nontransgenic to 8 in the F5 transgenic lymph nodes.

We conclude from these results that in $H\text{-}2^b$ haplotype, positive selection of thymocytes carrying the F5 T-cell receptor occurs and this leads to skewing toward a $CD8^+$ phenotype reflecting the phenotype of the original clone from which the T-cell receptor was isolated. These results are consistent with previous studies in other TcR transgenic systems (Kisielow et al., 1988b; Sha et al., 1988b; Kaye et al., 1989).

### Effects of Expression of IE and Mtv Products in the Development of Thymocytes and T cells in F5 Transgenic Mice

The F5 T-cell receptor utilizes the Vβ11 segment of the β-chain gene family. T cells carrying the Vβ11 chain are normally clonally deleted in mice expressing a functional IE molecule and products from Mtv 8, 9, or 11 integrants (Dyson et al., 1991). In order to study the influence of these proteins on the development of Vβ11-bearing thymocytes, we bred the C57BL/10 F5 transgenic mouse to CBA/Ca ($H\text{-}2^k$; $IE^+$; Mtv $8^+$ and $9^+$) and to BALB/c ($H\text{-}2^d$; $IE^+$; Mtv $6^+$, $8^+$, and $9^+$) mice. F1 (bxk or bxd) mice were backcrossed to CBA/Ca or BALB/c, respectively, and bk, kk or bd, dd, and $F5^+$ progeny were identified as described in Materials and Methods and analyzed by three-color fluorometric analysis using monoclonal antibodies against CD4, CD8, and Vβ11. Figure 4A shows that the proportion of $CD8^+4^-$ thymocytes was reduced from 20% in bb mice to 4% in bk and 2% in kk F5 transgenic mice. The decrease, which was also observed in bd and dd mice (Fig. 4B), in which the proportion of $CD8^+4^-$ thymocytes was reduced to approximately 2%, is unlikely to be due to the absence of positive selection as the heterozygote (bk or bd) mice had just as few $CD8^+4^-$ thymocytes as the dd and kk animals, despite the presence of the $H\text{-}2^b$ positively selecting allele. The percentage of $CD4^+8^+$ double-positive thymocytes was increased slightly, but the actual cell number was not significantly affected.

In the periphery of bd and dd F5 transgenic mice, analysis of lymph node cells showed a reduction of $CD8^+4^-$ cells from 70% to 28% and 24%, respectively (Fig. 4B), whereas in lymph nodes of bk and kk mice, the reduction was less pronounced (Fig. 4A). Figure 4C represents the proportions of CD4$^+$8$^-$ or CD4$^-$8$^+$ cells found in the different MHC haplotype mice.

### Expression of V$\beta$11 on Thymocytes and T Cells in F5 Transgenic Mice Expressing IE and Mtv Products

Thymocytes and peripheral T cells from F5 transgenic mice of bk, kk, bd, and dd haplotypes were examined by three-color fluorometric analysis using monoclonal antibodies against CD4, CD8, and V$\beta$11.

Figure 5 shows such an analysis comparing the V$\beta$11 levels on thymocytes from a bb F5 transgenic mouse to those found on thymocytes from a bk F5 transgenic mouse. Thymocytes from the bk mice had lost the intermediate level of V$\beta$11 expression and most thymocytes were expressing low levels. The level of V$\beta$11 expression on the CD4$^+$8$^+$ was unaffected, whereas most of the single-positive CD4$^+$8$^-$ or CD4$^-$8$^+$ populations express no or low levels of V$\beta$11, with the CD4$^+$8$^-$ population being most affected.

In the peripheral lymphoid tissues of F5 transgenic mice expressing IE and Mtv products, mature T cells that had low or very low levels of V$\beta$11 on their surface predominated (Fig. 5A). However, in F5 transgenic mice of H-2$^{bk}$ or H-2$^{kk}$ haplotype, a proportion of CD8$^+$ cells persists that have V$\beta$11$^{int}$ levels similar to those observed in the bb F5 TcR transgenic mice. No expression of V$\beta$11 was observed in the double-negative

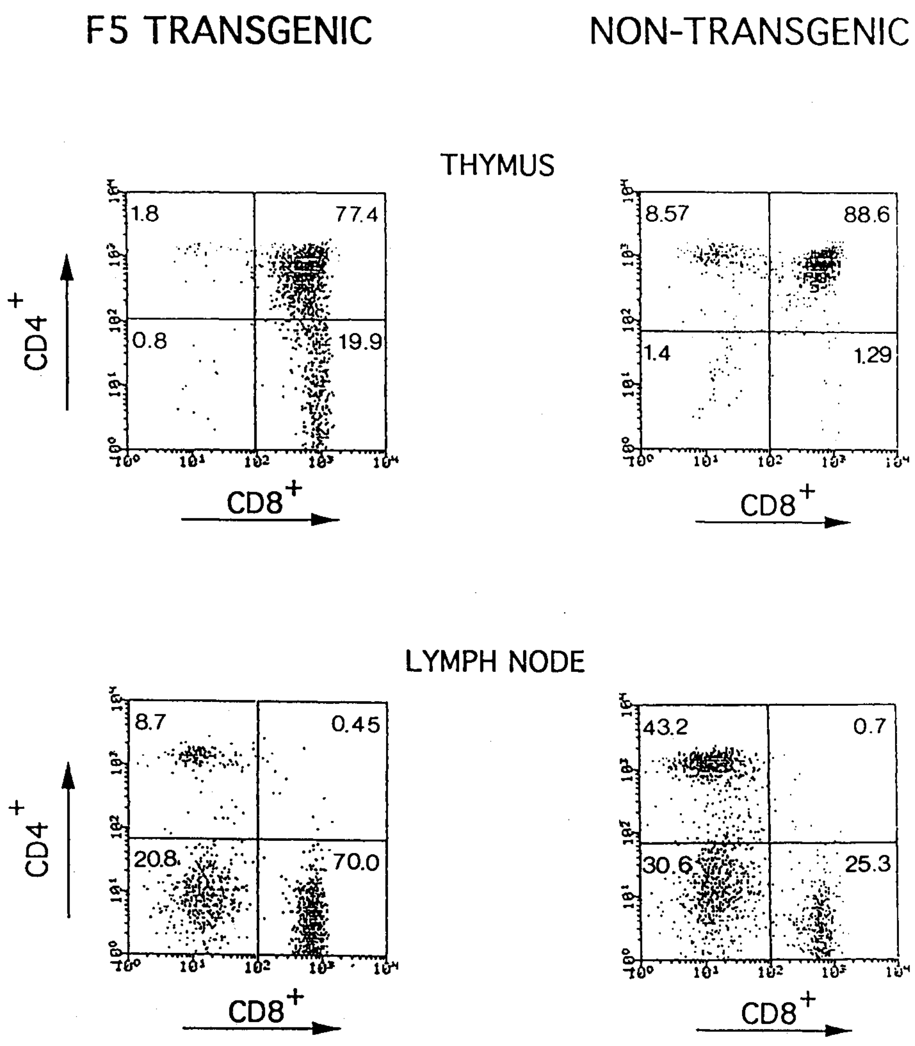

FIGURE 3. Positive selection in F5 transgenic mice. Thymocytes and lymph node cells of F5 and non-transgenic mice were stained with anti-CD4 and anti-CD8 antibodies and the proportion of the different subpopulations was determined.

CD4⁻8⁻ population in the lymph node cells of these mice (Fig. 5B).

We conclude from these results that expression of IE molecules in association with endogenous Mtv products leads to clonal deletion of V$\beta$11-bearing, Mtv-reactive cells at the late double-positive stage. Most of the cells that escape to the periphery appear to have downregulated their expression of V$\beta$11 or had low levels of V$\beta$11 originally.

### Functional Properties of T Cells from F5 Transgenic Mice

In order to assess the ability of the T cells from the F5 transgenic mice to recognize the cognate peptide on appropriate target cells, cytotoxic assays were performed, as described in Materials and Methods. From Table 1, it can be seen that the presence of IL-2 in the culture medium is required for the generation of peptide-specific cytotoxic T cells from F5 transgenic mice. Spleen cells taken directly from mice were not cytotoxic (data not shown and Mamalaki et al., 1992) unless mice had been injected with peptide *in vivo* (Mamalaki et al., 1992). Cultures generating peptide-specific cytotoxic T cells showed development of blast cells from day 2 or 3 after culture: Assays for cytotoxicity were generally performed on day 5, but cytotoxicity could be seen from day 4 to day 7.

Cultures stimulated with peptide-pulsed B10

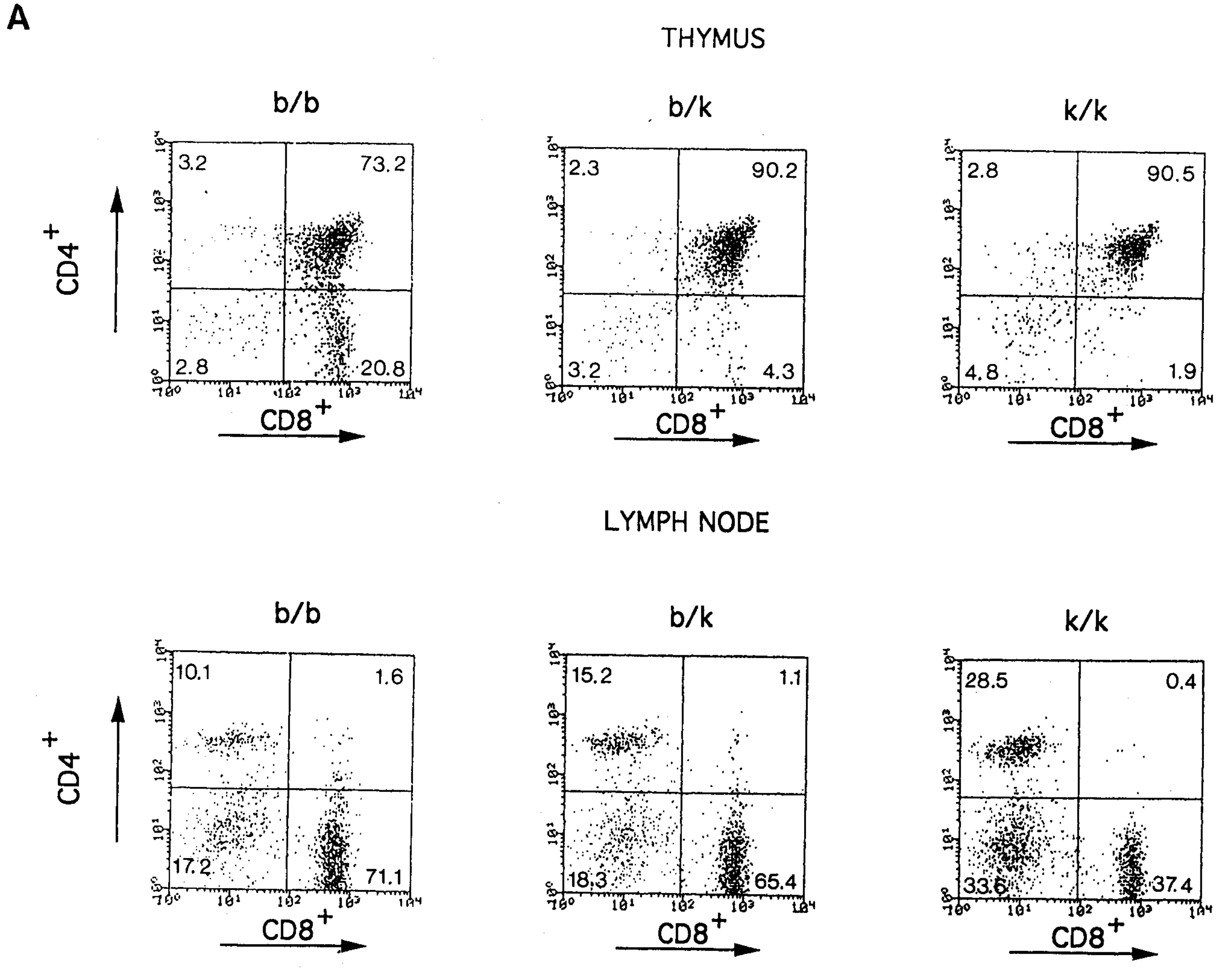

FIGURE 4. Deletion of mature transgenic lymphocytes in the presence of IE and endogenous superantigens. (A) Thymocytes and lymph node cells from transgenic animals of H-$2^{bb}$, H-$2^{bk}$, and H-$2^{kk}$ haplotypes were stained as described in Fig. 2 and the patterns of expression of CD4 and CD8 are shown.

splenic cells (B10$^P$) in the absence of IL-2 showed no evidence of proliferation or differentiation into cytotoxic effector cells. Percentage recovery of cells (a measure of proliferation) from cultures containing IL-2 but no peptide-pulsed B10 splenic cells (B10$^P$) was lower than those with both IL-2 and B10$^P$, but levels of cytotoxicity were comparable, or only slightly lower (Table 2). Percentage recoveries of cells from cultures with IL-2 and B10$^P$ were always higher than those with B10 spleen cells not pulsed with peptide (B10) (Table 1).

Despite the thymic deletion or failure to positively select CD8$^+$ V$\beta$11$^+$ T cells in bk or kk mice (see Fig. 4), their peripheral T cells could generate peptide-specific cytotoxic T cells after *in vitro* culture in the presence of IL-2 (Table 2). In the case of spleen cells from kk mice, the inclusion of B10$^P$ in the culture did not increase percentage recoveries and tended to increase the level of nonspecific alloreactive lysis of EL-4 cells (Table 2).

### T Cells Bearing the Transgenic T-Cell Receptor Accumulate in F5 Scid Transgenic Mice

The presence of T cells that can kill targets in an antigen-dependent manner in the periphery of F5

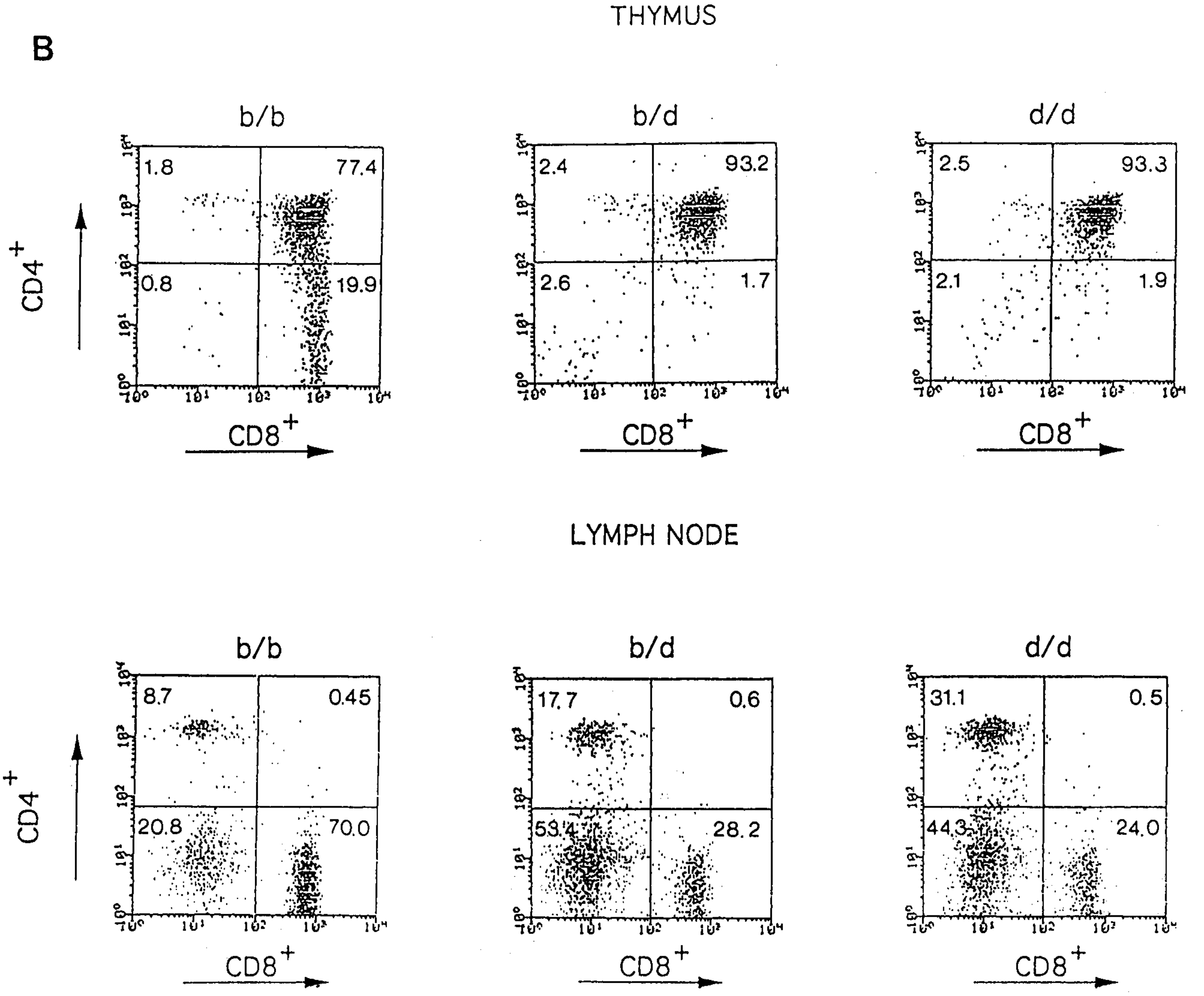

FIGURE 4. Deletion of mature transgenic lymphocytes in the presence of IE and endogenous superantigens. (B) Thymocytes and lymph node cells from transgenic animals of H-2$^{bb}$, H-2$^{bd}$, and H-2$^{dd}$ haplotypes were stained as described in Fig. 2 and the patterns of expression of CD4 and CD8 are shown.

transgenic mice of MHC bk, kk, bd, and dd haplotype indicated that these T cells may have been positively selected in the thymus using endogenously rearranged $\alpha$ chains that then pair with the transgenic V$\beta$11. Such cells could carry heterodimers of F5 $\alpha^{tg}\beta^{tg}$ type and random $\alpha^{end}\beta^{tg}$ type. It is possible that such cells would be selected on the $\alpha^{end}\beta^{tg}$ pair, but could kill targets in an antigen-dependent manner using the $\alpha^{tg}\beta^{tg}$. To test this possibility, F5 transgenic mice were backcrossed to BALB/c scid/scid mice. F5 mice homozygous for the scid mutation (scid/scid) and homozygous or heterozygous for H-2 (dd, db) were compared with F5 mice of scid/+ or +/+ and of dd or bd H-2 haplotype. Three-color fluorometric analysis showed similar subpopulation distribution to that shown in Fig. 4B, indicating no additional effect on the transgene phenotype of the scid mutation.

Table 3 shows the results of culturing spleen cells from F5 transgenic mice crossed and then backcrossed onto BALB/c scid/scid mice. There was no difference in the ability of bd and dd F5 mice, either heterozygous or homozygous for the scid mutation, to generate peptide-specific cytotoxic T cells in culture.

These results appear to exclude the possibility that the T cells found in the periphery of bd, dd, bk, and kk mice have necessarily been positively selected using T-cell receptors that utilize endogenously rearranged $\alpha$- and $\beta$-chain genes, unless in the used scid/scid mice, the defect in

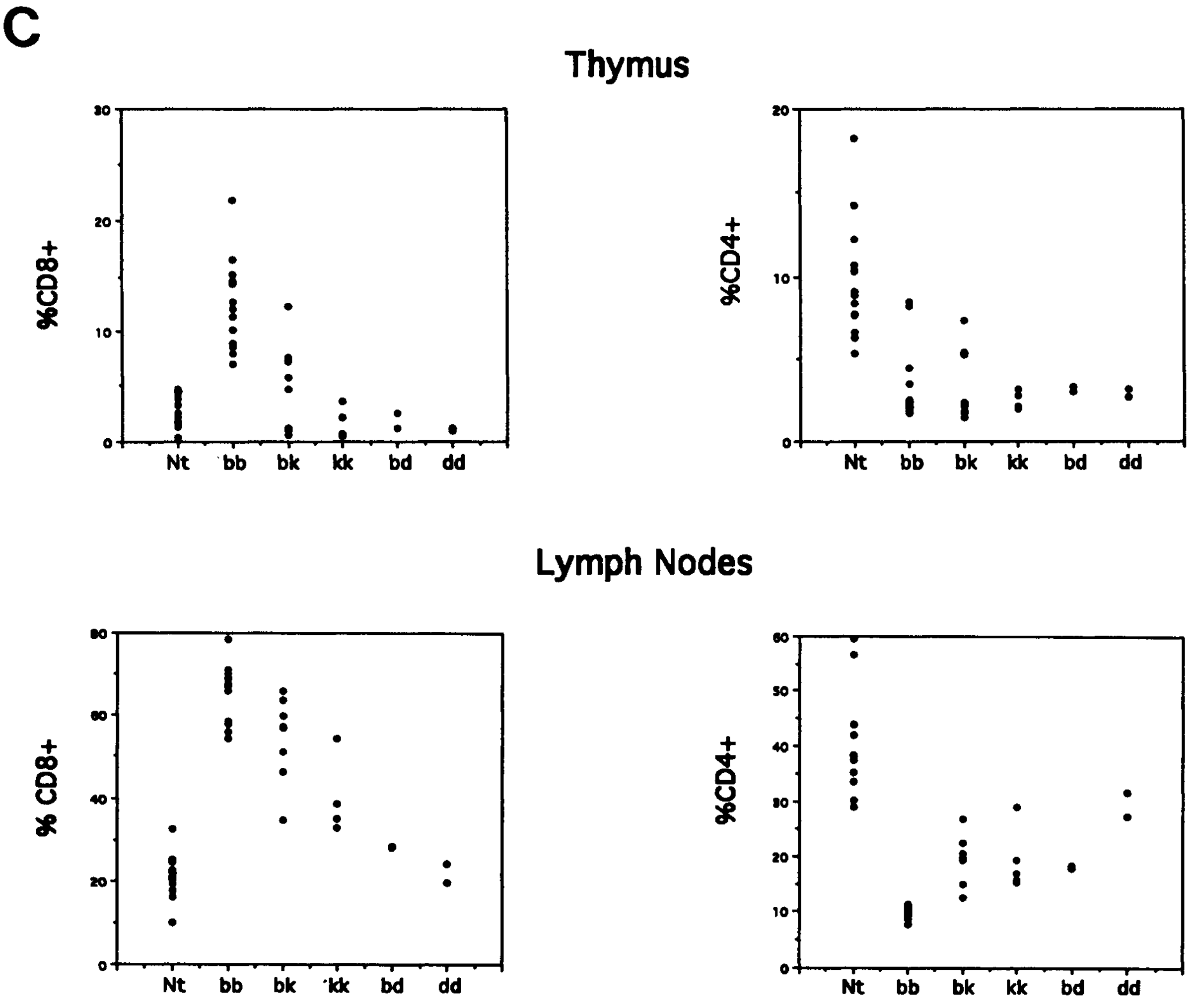

FIGURE 4. Deletion of mature transgenic lymphocytes in the presence of IE and endogenous superantigens. (C) Variability in proportion of $CD4^+8^-$ and $CD4^-8^+$ cells in thymus and lymph node of F5 transgenic mice of different MHC haplotypes.

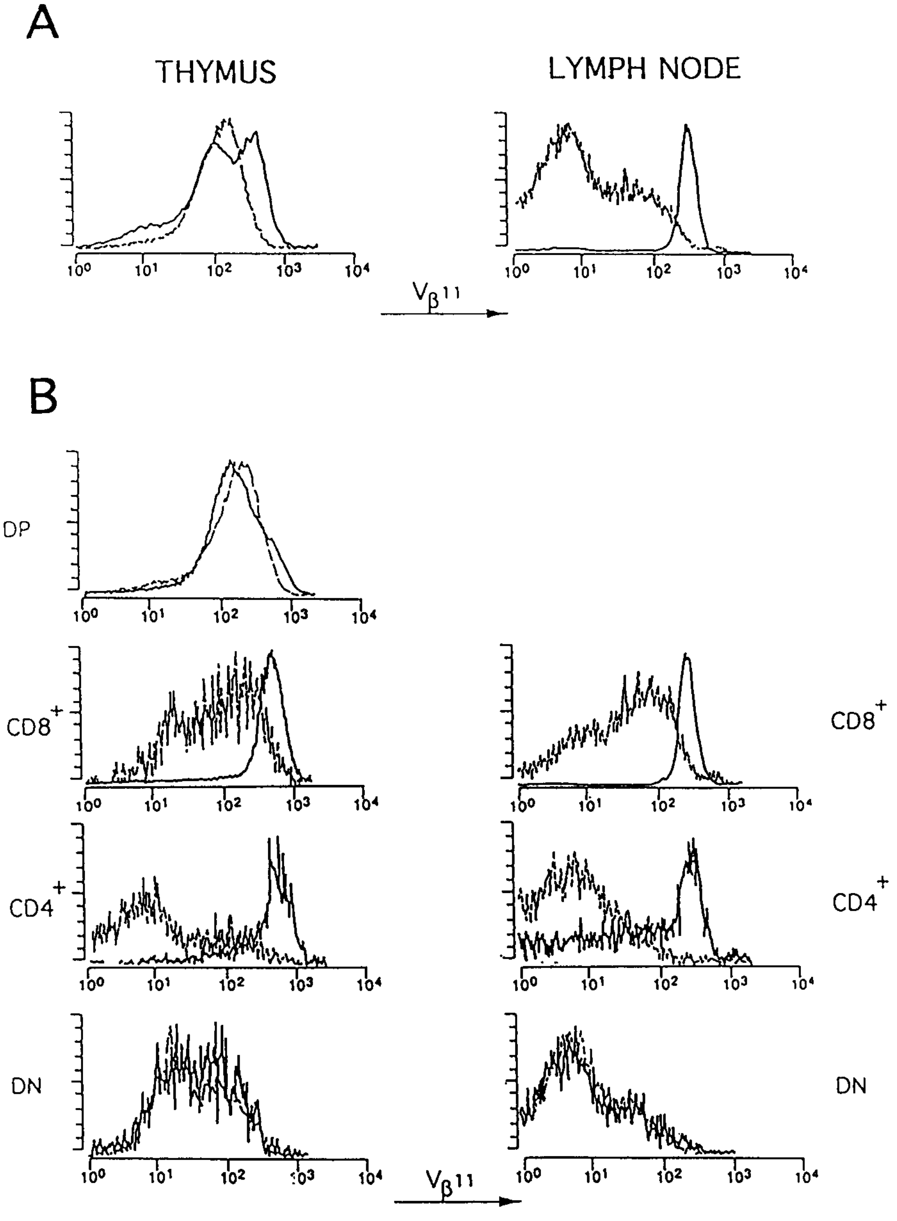

FIGURE 5. Downregulation of Vβ11 expression in the presence of IE and endogenous superantigens. (A) Expression of Vβ11 on total thymocytes and lymph node cells from F5 TcR transgenic mice of $H\text{-}2^{b/b}$ (solid lines) or $H\text{-}2^{b/k}$ (dashed lines) haplotype. (B) Expression of Vβ11 on the different subpopulations in thymocytes and lymph node cells from mice as described in Fig. 5A and stained as described in Fig. 2.

the rearrangement of the TcR loci is not complete.

### DISCUSSION

We have generated a mouse transgenic for a T-cell receptor (F5) specific for a peptide (αα366–374) of nucleoprotein of influenza virus in association with Class I MHC $D^b$. These mice show accumulation of single-positive $CD8^+$ lymphocytes in the thymus and in the periphery as a result of positive selection. Analysis of receptor expression using a monoclonal antibody that recognizes the Vβ11 chain of the F5 receptor showed that almost all $CD8^+$ cells (>90%) express the transgenic Vβ chain, whereas within the $CD4^+$ population only 70–90% of the cells express the transgenic Vβ11 in the thymus and 40–60% in the lymph nodes. It is possible that $CD4^+$ cells in the F5 transgenic mice are selected using endogenous α chains that can form functional dimers with the transgenic Vβ11 or other

TABLE 1
Conditions for Generation of Peptide-Specific Cytotoxic T Cells from F5 Transgenic Mice

| Experiment | Culture conditions | | | | | % lysis | |
|---|---|---|---|---|---|---|---|
| | No. responding cells | IL-2 | B10 | B10$^{P}$ | % recovery | EL-4 | EL-4$^{P}$ |
| 1 | 1x10$^{6}$ | – | – | + | 100 | 0 | 0 |
| | 1x10$^{6}$ | + | – | + | 225 | 2 | 62 |
| | 5x10$^{6}$ | – | – | + | 90 | 0 | 0 |
| | 5x10$^{6}$ | + | – | + | 330 | 3 | 66 |
| 2 | 1x10$^{6}$ | – | – | + | 95 | 0 | 0 |
| | 1x10$^{6}$ | + | – | – | 95 | 0 | 22 |
| | 1x10$^{6}$ | + | + | – | 95 | 0 | 29 |
| | 1x10$^{6}$ | + | – | + | 330 | 0 | 42 |
| 3 | 2x10$^{6}$ | + | + | – | 50 | 19 | 54 |
| | 2x10$^{6}$ | + | – | + | 93 | 5 | 57 |

TABLE 2
Function of F5 Transgenic Responder Spleen Cells from F5 bb, bk, and kk Mice

| Experiment | Responder | Culture conditions | | | % lysis | |
|---|---|---|---|---|---|---|
| | | IL-2 | B10$^{P}$ | % recovery | EL-4 | EL-4$^{P}$ |
| 1 | bb | + | + | 400 | 19 | 54 |
| | | + | – | 93 | 20 | 39 |
| | bk | + | + | 240 | 22 | 54 |
| | | + | – | 120 | 20 | 38 |
| | kk | + | + | 140 | 33 | 55 |
| | | + | – | 120 | 16 | 36 |
| 2 | bb | + | + | 120 | 8 | 52 |
| | | + | – | 110 | 9 | 35 |
| | bk | + | + | 140 | 11 | 46 |
| | | + | – | 90 | 14 | 46 |
| | kk | + | + | 120 | 20 | 46 |
| | | + | – | 130 | 9 | 39 |

TABLE 3
Peptide-Specific CTL from Spleens of scid/+ and scid/scid F5 Transgenic Mice

| Responder | | Culture conditions | | % lysis | |
|---|---|---|---|---|---|
| H-2 | scid | IL-2 | % recovery | EL-4 | EL-4$^{P}$ |
| bd | scid/+ | + | 112 | 13 | 52 |
| dd | scid/+ | + | 45 | 13 | 62 |
| bd | scid/scid | + | 62 | 12 | 69 |
| dd | scid/scid | + | 50 | 5 | 60 |

endogenous V$\beta$ chains, which have escaped allelic exclusion, thus generating Class II MHC restricted T cells that are subsequently positively selected.

Spleen cells from the F5 transgenic mice can be stimulated *in vitro* to differentiate into effector cells that kill target cells in an antigen-dependent manner. This differentiation is dependent on the presence of IL-2 in the culture medium during a 4–5 day culture period. F5 T cells do not replicate or die in culture in the absence of IL-2 (Table 1) and do not become effector cells in the protocol described in this paper. It is unclear at the moment whether the peripheral T cells in F5 C57BL/10 mice are in an anergic state and, if so, why.

The F5 T-cell receptor utilizes the V$\beta$11 member of the TcR$\beta$ gene family. Most cells carrying the V$\beta$11 chain are clonally deleted in mice that express a functional Class II MHC IE $\alpha\beta$ heterodimer and certain endogenous Mtv genes. In order to study the effects of such deleting elements on the development and function of F5 transgenic lymphocytes, we bred the transgenic F5 (C57BL/10 H-2$^{b}$) mice to CBA/Ca H-2$^{k}$ or BALB/c H-2$^{d}$ backgrounds. F1 mice and their backcrosses to CBA/Ca and BALB/c F2 mice were analyzed for T-cell development and their ability to kill target cells in a peptide-dependent manner. Phenotypic analysis showed that the number of mature single-positive CD8$^{+}$ cells carrying the V$\beta$11 TcR chain are reduced in kb, kk, db, and dd mice. We conclude from this that most of the mature V$\beta$11-bearing lymphocytes are clonally deleted in these mice in an effort to establish tolerance to endogenous superantigens. As in normal H-2 IE$^{+}$ mice with Mtv 8, 9, or 11 genomes (Dyson et al., 1991), a small percentage of V$\beta$11 cells escape deletion and appear in the periphery. The remaining V$\beta$11$^{+}$ CD8$^{+}$ or V$\beta$11$^{+}$ CD4$^{+}$ cells found in the thymus and the periphery have reduced levels of transgenic TcR.

Thus, in this mouse model, both clonal deletion as evidenced by the absence of single-positive cells and possibly downregulation of the T-cell receptor levels are used to establish and maintain functional tolerance.

This differs from the findings in adult F5 transgenic mice injected intraperitoneally with the cognate peptide. Under these conditions, it is the double-positive cells that are depleted in the thymus. This difference could be explained by levels of presented antigen: Mtv products are in lower abundance than the levels of administered peptide. Such a hypothesis would predict that sufficiently low levels of administered peptide would eventually deplete the thymus from single-positive cells leaving the double-positive population intact. However, in titration experiments of peptide dose, we failed to observe this phenomenon. Instead, the extent of depletion of the double-positive population was dependent on peptide concentration, with no effect seen using 0.25 nmol/g weight. Alternatively, peptide injection leads to presentation of the antigen by all cells expressing MHC, whereas Mtv products may be synthesized in a subpopulation of antigen-presenting cells. The mechanisms underlying this difference are under investigation in our laboratory at the moment. These results are reminiscent of the findings by Pircher and colleagues. In that model, mice transgenic for a TcR specific for lymphocytic choriomeningitis virus (LCMV) and mixed lymphocyte stimulatory (Mls) antigen were examined for tolerance to these two antigens. Thus, mice tolerant to LCMV have low numbers of $CD4^{+}8^{+}$ thymocytes, whereas mice tolerant to Mls show deletion of single-positive mature cells (Pircher et al., 1989).

Functional studies on spleen cells from bk, kk, bd, or dd mice showed that such circulating lymphocytes can be stimulated to kill target cells in an antigen-dependent manner. Thus, the mature T cells found in these mice carry a potentially functional receptor, but are probably kept in an anergic or quiescent state within the mouse until they are cultured *in vitro* in the presence of IL-2.

There have been a number of examples of potentially self-reactive T cells that fail to be activated *in vivo* but can be activated *in vitro* in the presence of IL-2 (Essery et al., 1988; Beverly et al., 1992). A similar mechanism may operate in the case of the bk, kk, bd, and dd $F5^{+}$ mice in which IL-2 addition to cultures may cause a reversal of the *in vivo* anergic state of these lymphocytes. Experiments addressing the ability for response to antigen *in vivo* in these mice are currently under way.

The circulating T cells in these mice bearing a potentially functional transgenic receptor might arise by positive selection of T cells that have rearranged endogenous $\alpha$-chain genes that can form positively selectable T-cell receptors in association with the transgenic $V\beta11$. Spleen cells from these mice would contain T cells that bear two $\alpha$ chains and that would be capable of killing target cells in an antigen-dependent manner, if properly stimulated. In order to investigate this possibility, we backcrossed the F5 TcR transgenic mice to scid/scid BALB/c mice until we obtained scid/scid, $F5^{+}$, $H\text{-}2^{bd}$ or scid/scid, $F5^{+}$, $H\text{-}2^{dd}$ mice. Because the scid/scid genotype ensures impaired, if not entirely abolished, ability to rearrrange productively the TcR $\alpha$ and $\beta$ loci, T cells appearing in these mice would carry obligatorily the transgenic $\alpha\beta$ dimer. Indeed, the thymus develops normally in scid/scid, $F5^{+}$, $H\text{-}2^{dd}$ mice with the expected clonal deletion of most of the mature single-positive populations because of endogenous Mtv-derived superantigens of BALB/c and C57BL/10 background. To our surprise, in the periphery of these mice, T cells of $CD4^{+}$ or $CD8^{+}$ phenotype carrying the transgenic receptor are present, albeit in lower numbers. Therefore, the presence of endogenous $\alpha$ chains that rescue these cells in an $IE^{+}$ $Mtv^{+}$ background seems unlikely, but cannot be excluded at the moment, because productive rearrangements can occur to a certain degree even in scid/scid mice. The mature T cells in these mice appear to be capable of differentiating in the presence of IL-2 to kill target cells in an antigen-dependent manner. We are left with only a few explanations for this phenomenon. One would entail the presence of a cross-reacting, positively selecting element in BALB/c background that allows the maturation of some cells. The fact that we obtained similar results using two different H-2 haplotypes argues against such an explanation, but cannot be excluded at present. Alternatively, clonal deletion of the reactive $V\beta11^{+}$ T cells by the k or d haplotypes is inefficient due to the sheer numbers of thymocytes bearing this receptor. Those that escape in the periphery appear to be unreactive to endogenous IE and Mtv products due to downregulation of the transgenic T-cell receptor levels. These cells, however, can be triggered *in*

*vitro* by appropriate lymphokines. Finally, it is not entirely unlikely that the cells do not need to pass through a positive selection stage if they carry a rearranged functional T-cell-receptor gene complement. Such receptor would have been positively selected for its usefulness during its original appearance in the influenza immunized mouse from which the F5 cytotoxic T-cell clone was isolated. In the F5 TcR transgenic mouse, this would imply that positive selection is not a change in the metabolism or state of the developing thymocyte, simply that positive selection is the result of a TcR engagement in a way that does not lead to deletion.

### MATERIALS AND METHODS

### Gene Construct and Transgenic Mice

The cDNAS for the $\alpha$ and $\beta$ chains of the F5 T-cell receptor were cloned in the 5' untranslated region of the human CD2 mini gene (Greaves et al., 1989). A human CD2 minigene that carries a frameshift mutation in its coding region was partially digested with SacI. The linearized fragment at the SacI site in the first exon was isolated, treated with S1 nuclease, and the ends were blunted using the Klenow fragment of DNA polymerase I. A unique synthetic EcoRI site was cloned into the blunted SacI site. The artificial EcoRI was blunt-ended and the cDNAs for the $\alpha$ and $\beta$ chains of the F5 T-cell receptor (Palmer et al., 1989) were inserted in this position.

The chimeric F5$\alpha$ and F5$\beta$ constructs were coinjected into C57BL/10 embryos as previously described (Lang et al., 1988). The transgenic animals were crossed with either C57BL/10, CBA/Ca, BALB/c, or scid/scid mice. H-$2^{bk}$, H-$2^{kk}$, H-$2^{bd}$, or H-$2^{dd}$ haplotypes of F5 transgenic mice were determined by Southern blot analysis using a 0.68-kb BamHI fragment 5' to the H-2Kb gene (Weiss et al., 1984). F5 transgenic mice were typed scid/scid by quantitating serum immunoglobulin levels. Preparation of DNA and RNA and analysis by Southern or Northern blot were carried out as described (Lang et al., 1988).

### Flow Cytometry

For three-color analysis, $10^6$ thymocytes or lymph node cells were stained with FITC-conjugated anti-CD8 (53-6.7), (Becton & Dickinson), anti-CD4-RED613 (YTS 191.1.2) (Gibco BRL), and biotinylated KTII anti-V$\beta$11 antibody (kind gift from Kyuhei Tomonari, CRC) (Tomonari and Lovering, 1988), followed by phycoerythrin-conjugated streptavidin (Biogenesis).

### *In vitro* Cultures

Spleens were removed aseptically from F5 transgenic mice, their nontransgenic littermates, and nontransgenic C57BL/10 mice. The ages of mice used ranged from 3 to 8 weeks. Spleens were teased into cell suspensions in balanced salt solution (BSS) containing 2% fetal calf serum (FCS), filtered through nylon mesh to remove cell clumps, and then centrifuged. Pellets of spleen cells to be used as a source of antigen were then treated to remove red blood cells, using brief exposure to distilled water followed by restoration to isotonicity with 10×BSS and then centrifuged. Cell pellets were resuspended in complete RPMI medium (RPMI 1640 with 10% FCS, 10 mM Hepes of pH 7.2, $5\times10^{-5}$ M 2-mercaptoethanol, glutamine, penicillin, and streptomycin) and counted. An appropriate number of cells to be used as antigen were sensitized with influenza nucleoprotein peptide (NP 366-379) by adding 10 $\mu$l of peptide at 100 $\mu$M per $10^7$ cells to the cell pellet. They were then incubated at 37°C for 45 min, washed twice in complete RPMI medium, resuspended at an appropriate concentration, and irradiated before addition to cultures. Spleen cells to be used as responders did not have red blood cells removed, but were suspended in complete RPMI medium, counted, and adjusted to an appropriate concentration for culture.

### Cytotoxic T-cell Assays

These were carried out using as targets EL-4 cells growing in log phase *in vitro:* $2\times10^6$ EL-4 were labeled for 60 min at 37°C in 0.1 ml BSS medium containing 100 $\mu$Ci $^{51}$Cr sodium chromate: To a pellet of another aliquot of $2\times10^6$ EL-4 was added 100 $\mu$l of 100 $\mu$M influenza nucleoprotein peptide NP 366-379 together with 100 $\mu$Ci $^{51}$Cr sodium chromate and incubation carried out for 60 min at 37°C. After incubation, both aliquots of EL-4 were washed twice and then resuspended in complete RPMI medium at $1\times10^5$/ml before dispensing 100 $\mu$l ($1\times10^4$) into each microtitre well to which effector T cells had been added. Effector cells were harvested from spleen cells of transgenic mice and cultured at 37°C in a 5% $CO_2$ atmosphere for 5 days in the presence of 50 IU/ml rIL-2 with or without C57BL/10 spleen cells sensitized or not with NP 366-379 peptide, as described before. Effector cells were harvested, centrifuged, and resuspended in fresh complete RPMI medium, counted, and then volume adjusted to give a concentration of $3\times 10^6$/ml. Each effector cell suspension was dispensed in round-bottomed microtitre wells in a volume of 100 $\mu$l in triplicate and then four serial, one in three dilutions, were carried out. After addition of $1\times10^4$ target cells/well, effector-to-target-cell ratios of 30:1, 3:1, and 1:1 were present. The assay plates were briefly centrifuged and then incubated for 3 hr at 37°C in a 5% $CO_2$ atmosphere. 100 $\mu$l of supernatant was then harvested from each plate and counted for $^{51}$Cr. The percent specific lysis was calculated according to the formula:

$$\%\ \text{specific lysis}=(E-C/M-C)\times100$$

where $E$ is the cpm from wells with effectors present, C is the cpm from control wells with target cells incubated in medium alone, and M is the maximum released counts from target cells incubated with 5% Triton-100. Twelve-point regression analysis was performed for each titration curve and the % lysis at an effector:target ratio of 10:1 was taken from this curve. Significant positive lysis was taken as levels over 10% specific lysis from curves where the $r^2$ value lay between 0.80 and 1.00. These are underlined in the tables.

*(Received November 3, 1992)*

*(Accepted January 4, 1993)*

### REFERENCES

Adkins B., Mueller C., Okada C.X., Reichert R A., Weissman I.L., and Spanngrude G.J.W. (1987). Early events in T cell maturation. Ann. Rev. Immunol. **5:** 325–365.

Berg L.J., Pullen A.M., Fazekas de St. Grot B., Mathis D., Benoist C., and Davis M.M. (1989) Antigen/MHC-specific T cells are preferentially exported from the thymus in the presence of their MHC ligand. Cell **58:** 1035–1046.

Bevan M.J., and Fink P. (1978). The influence of thymus H-2 antigens on the specificity of maturing killer and helper cells. Immunol. Rev. **42:** 3–19.

Beverly B., Kang S.-M., Lenardo M.J., and Schwarz R.H. (1992). Reversal of *in vitro* T cell clonal anergy by IL-2 stimulation. Int. Immunol. **4:** 661–671.

Blackman M., Kappler J., and Marrack P. (1990). The role of the T cell receptor in positive and negative selection of developing T cells. Science **248:** 1335–1341.

Blackman M.A., Marrack P., and Kappler J. (1989). Influence of the major histocompatibility complex on positive thymic selection of V$\beta$17a$^+$ T cells. Science **244:** 214–217.

Davis M.M., and Bjorkman P.J. (1988). T-cell antigen receptor genes and T-cell recognition. Nature **334:** 395–402.

Dyson P.J., Knight A.M., Fairchild S., Simpson E., and Tomonari K. (1991). Genes encoding ligands for deletion of V$\beta$11 T cell cosegregate with mammary tumour virus genomes. Nature **349:** 531–532.

Essery G., Feldmann M., and Lamb J.R. (1988). Interleukin-2 can prevent and reverse antigen-induced unresponsiveness in cloned human T lymphocytes. Immunology **64:** 413–417.

Greaves D.R., Wilson F., Lang G., and Kioussis D. (1989). Human CD2 3'-flanking sequences confer high-level T-cell specific position independent gene expression in transgenic mice. Cell **56:** 979–986.

Kappler J.W., Roehm N., and Marrack P. (1987). T-cell tolerance by clonal elimination in the thymus. Cell **49:** 273–280.

Kappler J.W., Staerz U., White J., and Marrack P. (1988). Self-tolerance eliminates T-cells specific for Mls-modified products on the major histocompatibility complex. Nature **332:** 35–40.

Kaye J., Hsu M.L., Sauron M.E., Jameson S.C., Gascoigne N.J., and Hendrick S.M. (1989). Selective development of CD4$^+$ T cells in transgenic mice expressing a class II MHC-restricted antigen receptor. Nature **341:** 746–749.

Kisielow P., Bluthman H., Staerz V.D., Steimetz M., and von Boehmer H. (1988a). Tolerance in T cell receptor transgenic mice involves deletion of non mature CD4$^+$CD8$^+$ thymocytes. Nature **333:** 742–746.

Kisielow P., Teh H.S., Blutmann H., and von Boehmer H. (1988b). Positive selection of antigen-specific T cells in thymus by restricting MHC molecules. Nature **335:** 730–733.

Lang G., Wotton D., Owen M.J., Sewell W.A., Brown M.H., Mason D., Crumpton M.J., and Kioussis D. (1988). The structure of the human CD2 gene and its expression in transgenic mice. EMBO J. **7:** 1672–1682.

Loh D.Y. (1991). Molecular requirements for cell fate determination during T-lymphocyte development. New Biologist **3:** 924–932.

MacDonald H.R., Schneider R., Lees R.K., Howes R.C., Acha-Orbea H., Festenstein H., Zingernagel R.M., and Hengartner H. (1988). T-cell receptor V$\beta$ use predicts reactivity and tolerance to Mls-encoded antigens. Nature **332:** 40–45.

Mamalaki C., Norton T., Tanaka Y., Townsend A.R., Chandler P., Simpson E., and Kioussis D. (1992). Thymic depletion and peripheral activation of class I major histocompatibility complex-restricted T cells by soluble peptide in T-cell receptor transgenic mice. Proc. Natl. Acad. Sci. USA **89:** 11342–11346.

Matzinger P., and Guerder S. (1989). Does T-cell tolerance require a dedicated antigen-presenting cell. Nature **338:** 74–76.

Mazda O., Watanabe Y., Gyotoku J.-I., and Katsura Y. (1991). Requirement of dendritic cells and B cells in the clonal deletion of Mls-reactive T cells in the thymus. J. Exp. Med. **173:** 539–547.

Ohashi R.S., Pircher H., Burki K., Zinkernagel R.M., and Hengartner H. (1990). Distinct sequence of negative or positive selection implied by thymocyte T-cell receptor densities. Nature **346:** 861–863.

Palmer M.S., Bentley A., Gould K., and Townsend A.R.M.

(1989). The T cell receptor from an influenza-A specific murine CTL clone. Nuc. Acid. Res. **17:** 2353.

Pircher H., Burki K., Lang R., Hengartner H., and Zingernagel R.M. (1989). Tolerance induction in double specific receptor transgenic mice varies with antigen. Nature **342:** 559–561.

Robey E.A., Ramsdell F., Gordon J.W., Mamalaki C., Kioussis D., Youn H.Y., Gottlieb P.D., Axel R., and Fowlkes B.J. (1992). Int. Immunol. **4:** 969–974.

Schwartz R.H. (1989). Acquisition of immunologic self-tolerance. Cell **57:** 1073–1081.

Scott B., Bluthmann H., Teh H.S., and von Boehmer H. (1989). The generation of mature T cells requires interaction of the $\alpha\beta$ T-cell receptor with major histocompatibility antigens. Nature **338:** 591–593.

Sha W.C., Nelson C.A., Newberry R.D., Kranz D.M., Russel J.H., and Loh D.Y. (1988a). Selective expression of an antigen receptor on CD8-bearing T lymphocytes in transgenic mice. Nature **335:** 271–274.

Sha W.C., Nelson C.A., Newberry R.D., Kranz D.M., Russel J.H., and Loh D.Y. (1988b). Positive and negative selection of an antigen receptor on T cells in transgenic mice. Nature **336:** 73–76.

Speiser D.E., Lees R.K., Hengartner H., Zingernagel R.M., and MacDonald H.R. (1989). Positive and negative selection of T cell receptor V$\beta$ domains controlled by distinct cell populations in the thymus. J. Exp. Med. **170:** 2165–2170.

Sprent, J., Lo D., Gao E.K., and Ron Y. (1988). T cell selection in the thymus. Immunol. Rev. **101:** 173–190.

Sprent J.H., von Boehmer H., and Nabholz M. (1975). Association of immunity and tolerance to host H-2 determinants in irradiated $F_1$ hybrid mice reconstituted with bone marrow cells form one parental strain. J. Exp. Med. **142:** 321–331.

Teh H., Kishi H., Scott B., and von Boehmer H. (1989). Deletion of autospecific T cells in T cell receptor (TcR) transgenic mice spares cells with normal TcR levels and low levels of CD8 molecules. J. Exp. Med. **169:** 795–806.

Tomonari K. (1992). Positive selection of V$\beta2^+$ CD8$^+$ T cells. Int. Immunol. **4:** 1195–1198.

Tomonari K., and Lovering E. (1988). T-cell receptor specific monoclonal antibodies against a V$\beta$11-positive mouse T-cell clone. Immunogenetics **28:** 445–451.

Townsend A.R.M., Rothbard J., Gotch F.M., Bahadur G., Wraith D., and McMichael A.J. (1986). The epitopes of influenza nucleoprotein recognized by cytotoxic lymphocytes can be defined with short peptides. Cell **44:** 959–968.

von Boehmer H. (1990). Developmental biology of T cells in T-cell receptor transgenic mice. Ann. Rev. Immunol. **8:** 531–556.

Weiss E.H., Golden L., Fahrner K., Mellor A.L., Devlin J.J., Bullman H., Tiddens H., Bud H., and Flavell R.A. (1984). Organization and evolution of the Class I gene family in the major histocompatibility complex of the C57BL/10 mouse. Nature **310:** 650–655.

